# Effectiveness of mindfulness-based stress reduction on depression, anxiety, and stress of women with the early loss of pregnancy in southeast Iran: a randomized control trial

**DOI:** 10.1186/s12978-022-01543-2

**Published:** 2022-12-29

**Authors:** Masumeh Nasrollahi, Masumeh Ghazanfar Pour, Atefeh Ahmadi, Mogaddameh Mirzaee, Katayoun Alidousti

**Affiliations:** 1 Student Research Committee, Department of Midwifery, Razi Faculty of Nursing and Midwifery, Kerman University of Medical Sciences, Kerman, Iran; 2 Department of Midwifery, Razi Faculty of Nursing and Midwifery, Kerman University of Medical Sciences, Kerman, Iran; 3 Department of Guidance and Counselling/Medical Practitioner, Nursing Research Center, Razi Faculty of Nursing and Midwifery, Kerman University of Medical Sciences, Kerman, Iran; 4 Department of Biostatistics Modeling in Health Research Center, Institute for Futures Studies in Health, Kerman University of Medical Sciences, Kerman, Iran; 5 Department of Midwifery, Neuroscience Research Center, Institute of Neuropharmacology, Kerman University of Medical Sciences, Kerman, Iran

**Keywords:** Mental health, Loss of pregnancy, Counseling, Mindfulness-based stress reduction

## Abstract

**Background:**

The loss of the fetus may cause mental health problems in women. The present study aimed to determine the effect of mindfulness-based stress reduction (MBSR) on anxiety, depression, and stress in women with early pregnancy loss.

**Methods:**

This study was performed on 106 women with early pregnancy loss in Shiraz, Iran. The intervention group underwent eight counselling sessions. Pre-test and post-test were performed in both groups with the Depression, Anxiety, and Stress Scale (DASS) 21 questionnaire. Data were analyzed by SPSS 23.

**Results:**

There was a statistically significant difference between the mean scores in the intervention group *vs.* the control group in terms of anxiety (7.9 ± 1.07 *vs.* 13.79 ± 5.36, respectively), stress (9.26 ± 1.25 *vs.*18.13 ± 7.66, respectively), and depression (7.83 ± 1.05 *vs.*16.26 ± 11.06, respectively) (*P* < 0.0001).

**Conclusions:**

MBSR can be suggested to promote women's mental health.

## Plain language summary

Women who lose their pregnancies are more at risk than others for mental disorders such as anxiety, depression, and grief. Assessing stress, anxiety, and depression is critical to maintain women's overall health so that timely supportive interventions can be pursued if necessary. Eight MBSR intervention sessions were performed for 53 women with early pregnancy loss. The total intervention for all participants took 4 months. Results showed that the anxiety, depression, and stress of most participants decreased, which suggests that MBSR can be an effective non-pharmacological method to improve mental health.

## Introduction

Vaginal bleeding is a relatively common event that occurs in one-third of cases in the first trimester of pregnancy. It can increase the risk of premature rupture of the membranes and the onset of miscarriage, and it can be a sign of a pathological condition such as ectopic pregnancy, moles, or gestational trophoblastic disease [1]. Spontaneous abortion, moles, and ectopic pregnancy are adverse events that lead to depression and anxiety by affecting the quality of social life [2, 3]. Psychological complications in women with early pregnancy loss include PTSD, anxiety, and depression at rates of 28%, 32%, 16% one month and 38%, 20% and 5% three months later, respectively [4].

Kulathilaka et al*.* reported a prevalence of 26.6% for depression and 18.6% for depression in spontaneous abortion [5]. About 40% of miscarrying women have been found to be suffering from symptoms of grief shortly after miscarriage, and major depressive disorder has been reported in 10–50% of women after miscarriage [6]. Previous research has shown that 92% of women who experience pregnancy loss require high levels of psychological after-care, but only one-third of them receive this care as more attention is given to their physical needs [7]. Bilardi reported that in Australia, more than half of women (59%) were not offered any information about miscarriage or pregnancy loss support. Although almost all reported they would have liked some form of support, more than half (57%) did not receive follow-up care or emotional support [8].

Immediately after the early termination of pregnancy women experience disturbing dreams and sleep problems [9]. When bleeding causes a miscarriage, it usually causes significant emotional disturbance in marital satisfaction [1].

Assessing stress, anxiety, and depression is critical to maintaining women's overall health so that supportive interventions can be pursued if necessary. One type of non-pharmacological intervention is the MBSR program. The mindfulness approach, rooted in religious traditions such as those of Buddhism, is used as a behavioral intervention in clinical problems. First, Jan Kabat Zayn used it to treat chronic pain. Unlike other clinical approaches that emphasize changing unpleasant pressures, mindfulness increases a person's acceptance of undesirable phenomena and thus leads to resilience. MBSR is a counseling method that teaches a person to be present in the moment without worrying about the future and worrying about past events [10]. A study by Jazayeri et al*.* showed that MBSR may be suitable for people who do not want conventional therapies. In their study, the effectiveness of MBSR was shown to immediately reduce anxiety, stress, and depression and increase mental well-being after the last intervention [11]. Khajooyee also revealed in her study that MBSR reduced anxiety, depression, and stress in women with unwanted pregnancies [12].

The high prevalence of first trimester bleeding, the need for intervention to reduce related psychological problems, and the newness of the mindfulness counseling method led us to design a study aimed to determine the effect of mindfulness-based stress reduction (MBSR) on anxiety, depression, and stress of women with early loss of pregnancy.

## Methods

This randomized control trial aimed to determine the effect of MBSR on anxiety, depression, and stress in women who had inevitable first trimester miscarriage in Zeinabieh Hospital in Shiraz in 2020. Patients were recruited during the four months between August and November 2020.

Zeinabieh Hospital in Shiraz is a specialized women's hospital and is a referral center for women from other cities and even neighboring provinces.

The study's objectives were described to all women who came to the study site with bleeding leading to the termination of pregnancy in the first trimester of their pregnancy. If they met the inclusion criteria and were willing to participate in the study, written informed consent was obtained. According to a random allocation table, the participants were placed in the control or intervention group. Randomization was done by the block method. We considered the intervention group as "A" and the control group as "B", and then 27 blocks of four combinations of "A, B" were randomly selected using R statistical software version 3.2.1. A sequence of the letters A and B was produced, and each person who was referred to Zeinabieh Hospital was placed in either the intervention or control group based on the sequence produced.

Inclusion criteria included willingness to participate in the study, planned pregnancy, no history of known mental illness, no use of psychiatric drugs in the two months before the beginning of the study, and no event that would cause anxiety, depression, or stress.

Exclusion criteria included unwillingness to continue participation in the research, occurrence of an unfortunate incident that caused anxiety, stress, or depression during the study, and absence in more than one intervention session.

### Sample size

Based on a previous study [13], and 10% dropout:

$$\alpha =0.05, \quad 1-\beta =0.8$$

$${\sigma }_{1}=5, \quad {\sigma }_{2}=4.04, \quad d = 2.5$$

and the formula below:

$$n=\frac{{\left({z}_{1-\frac{\alpha }{2}}+{z}_{1-\beta }\right)}^{2}({\sigma }_{1}^{2}+{\sigma }_{2}^{2})}{{d}^{2}}$$

fifty-seven people were considered for each intervention and control group.

### Data collection tools

1. Demographic characteristics questionnaire collecting information about age, sex, level of education, occupation, blood type, willingness or unwillingness to become pregnant, history of infertility and IVF, number of pregnancies, number of children, history and type of previous deliveries, history of abortion, and hemoglobin level.
2. The DASS21 questionnaire (Depression, Anxiety, and Stress Scales)

The DASS21 questionnaire is an international and valid questionnaire that includes 21 questions that evaluates the three factors of anxiety, depression, and stress. Seven questions are assigned to each of the variables. The items are assessed on a 4-point Likert scale ranging from 0 to 3 (0 = “no” and 3 = “most of the time”). The intensity/frequency of the 4 points represent the degree to which participants have experienced each state in the last week. The subject’s total score in this questionnaire is within the range of zero to 63, and higher scores show higher stress, depression, and anxiety.

### Procedure

This study was approved by the Ethics Committee of Kerman University of Medical Sciences, Iran (ethics code No. Kmu.ac.ir.1398.217). Written informed consent was obtained as a requirement to enter the study and participants were able to withdraw from the study whenever they wanted. Unique codes were used for each of the participants to ensure information confidentiality.

After obtaining informed written consent, both groups performed a pre-test. Then intervention was performed in eight sessions of 2 h once a week for the intervention group. Interventions were performed in a counseling room that was quietly ventilated with suitable lighting and adequate space for practical exercises. Some daily exercises had to be done at home, and participants were trained to exercise 40 min every day. Participants were recommended to divide the 40 min into shorter sections and spread them throughout the day, integrating the exercises with their everyday activities to increase the awareness of their minds. Homework reminders were sent via text message. The intervention was done by a researcher who had taken a counseling course in midwifery and was also trained in mindfulness. Table 1 describes a summary of the counseling sessions.

**Table 1:** Summary of counseling sessions based on the MBSR approach for reduction of anxiety, depression, and stress in women with early pregnancy loss

Session	Content
1	Greeting and declaration of counseling rules, definition of the concepts of mindfulness and the main variables, description of the internal and external flow of the mind, Eating raisins, home works
2	Reviewing previous home works, mindful thinking, Mindful examination of body and sitting meditation, home works
3	Reviewing previous home works, focus on being present, practice seeing and hearing consciously in three minutes, focus on five senses in five minutes, home works
4	Reviewing previous home works, stress and the body's reaction, Practicing thoughts-emotions-body senses-behavior relationships, three minutes of concentration on an unpleasant event, mindful walking, home works
5	Reviewing previous home works, effective responses to stress, Three-Minute Breathing Space (3MBS), meditation in daily life, home works
6	Reviewing previous home works, conscious mind interactions, take care of yourself, practicing speaking and listening consciously, practicing consecutive thoughts in an hour, getting feedback from participants from practicing, presenting homework Homemade
7	Reviewing previous home works, being more careful, Mindful Yoga, making the unpleasant event enjoyable, providing homework
8	Reviewing previous home works, mountain meditation, summarization of all sessions, homework [14]

During the consultation, the control group received routine post-pregnancy care. The two groups completed the post-test questionnaire two weeks after the last session. After the post-test, a pamphlet containing a summary of the counseling sessions was provided to the control group for ethical considerations. During the study, four people in the intervention group and four people in the control group were excluded. One person was replaced due to re-pregnancy in the intervention group, and another person attended the meetings instead of her and three people refused to participate in more than two counseling sessions. Two people declined to answer the posttest in the control group. A third person was excluded because of participating in the psychological counseling sessions and another one because of re-pregnancy. Therefore, the original 57 participants in each group were reduced to 53 in both groups.

### Statistical analysis

Chi-square or Fisher tests were used to compare the intervention and control groups in terms of demographic variables and obstetric variables. The Mann–Whitney test was used to compare anxiety, depression, and stress between the intervention and control groups before the intervention. ANOVA test was used to compare these variables after the intervention, due to the significant difference in the "willingness to get pregnant again" variable between the intervention and control groups. Wilcoxon test was used to compare anxiety, depression, and stress before and after the intervention. Effect size was calculated using Cohen’s effect size formula.

## Results

The results showed that the mean age of women in the intervention and control groups was 28.93 ± 5.62 and 29.30 ± 6.32 respectively. Blood type O was seen in 37.7% of the intervention group and 47.3% of the control group. The hemoglobin level of most participants (53.75%) was 11–12 gr/dl. The majority of participants (96.25%) had no history of infertility. The cause of bleeding in most participants in both groups (60.4%) was abortion. The Chi-square and Fisher tests showed no statistically significant difference between the demographic variables and obstetric history of the two groups, and the two groups were homogeneous in the beginning of the study (Table 2).

**Table 2:** Comparison of distribution of demographic and midwifery variables of intervention and control group participants

Group	Intervention	Control	p-value
Variable	Mean ±SD	Mean ±SD	
Age	28.93± 5.62	±6.3229.3	0.81

Group	Intervention	Control	p-value	df	Test criteria
Variable	Frequency (%)	Frequency (%)			
Education					
Under diploma	18 (34%)	14 (26.4%)	0.54	2	1.2
Diploma	19 (35.8%)	18 (34%)			Chi-square
Academic	16 (30.2%)	21 (39.6%)			
Job					
Homemaker	42 (79.2%)	37 (69.8%)	0.26	1	1.24
Employee	11 (20.8%)	16 (30.2%)			Chi-square
Blood group type					
A	17 (32.1%)	14 (26.4%)	0.53	3	2.12
B	11 (20.8%)	12 (22.6%)			Fisher
AB	5 (9.4%)	2 (3.8%)			
O	20 (37.7%)	25 (47.2%)			
Hemoglobin					
<11	12 (22.6%)	11 (20.8)	0.83	2	0.35
11–12	27 (50.9%)	30 (56.6%)			Chi-square
>12	14 (26.4%)	12 (22.6%)			
Infertility history					
No	49 (92.5%)	53 (100%)	0.11	–	4.15
Yes	4 (7.5%)	0			Fisher
History of childbirth					
None	15 (28.3%)	7 (13.2%)	0.28	3	3.8
Cesarean	11 (20.8%)	15 (28.3%)			Chi-square
Vaginal delivery	22 (41.5%)	25 (47.2%)			
History of both cesarean section and natural delivery	5 (9.4%)	6 (11.3%)			
Number of previous pregnancy					
1	11(20.8%)	10 (18.9)	0/86	4	1/29
2	13(24.5%)	15 (28.3%)			Chi-square
3	16 (30.2%)	16 (30.2%)			
4	6 (11.3%)	8 (15.1%)			
5≤	7 (13.2%)	4 (7.5%)			
Number of previous live birth					
0	15 (28.3%)	13 (24.5%)	0.56	–	20.4
1	16 (30.2%)	22 (41.5%)			Fisher
2	18 (34%)	13 (24.5%)			
≥3	4 (7.5%)	5 (9.4%)			
Abortion history					
0	17 (32.1%)	16 (30.2%)	0.93	3	0.45
1	25 (47.2%)	24 (45.3%)			Chi-square
2	7 (13.2%)	7 (13.2%)			
≥3	4 (7.5%)	6 (11.3%)			
Gestational age(week)					
≤6	13 (24.5%)	9 (17%)	0.61	2	0.96
07–10	20 (37.7%)	23 (43.4%)			Chi-square
11–14	20 (37.7%)	21 (39.6%)			
Desire to get pregnant again					
No	19 (35.8%)	30 (56.6%)	0.03	1	4.59
Yes	34 (64.2%)	23 (43.4%)			Chi-square
History of bleeding in previous pregnancies					
No	39 (73.6%)	42 (79.2%)	0.49	1	0.47
Yes	14 (26.4%)	11 (20.8%)			Chi-square
History of Artificial fertility					
No	51 (96.2%)	53 (100%)	0.49	–	2.03
Yes	2 (3.8%)	0			Fisher
Reason of loss of pregnancy					
EP	13 (24.5%)	12 (22.6%)	0.95	2	0.09
Mole	8 (15.1%)	9 (17%)			Chi-square
Abortion	32 (60.4%)	32 (60.4%)			

The Mann–Whitney test showed that the mean scores of anxiety, depression, and stress in the two intervention and control groups before the MBSR intervention were not statistically significant, i.e., the two groups were homogeneous in these three variables (Table 3).

**Table 3:** Comparison of anxiety, depression, and stress in the two groups before and after the intervention

Variable	Anxiety		p-value	Depression		p-value	Stress		p-value	Total		p-value
	Mean±SD			Mean±SD			Mean±SD			Mean±SD		
Group	Before	After		Before	After		Before	After		Before	After	
Intervention	14.35±5.34	7.9±1.07	<0.0001***	15.11±6.34	7.83±1.05	<0.0001***	18.39±5.47	9.26±1.25	<0.001***	47.86±15.61	25±2.75	<0.0001***
Control	13.58±4.47	13.79±5.36	0.59***	14.47±5.96	16.26±11.06	0.02***	18.2±7.07	18.13±7.66	0.2***	46.26±15.19	48.18±19	0.35 *****
p-value	0.598*	<0.0001**		0.62*	<0.0001**		0.57*	<0.0001**		0.59****	<0.0001*	
Effect size		− 1.54	–		1.08-	–		− 1.63	–		− 1.5	–

*Mann–Whitney **ANCOVA ***Wilcoxon ****T test *****paired t test

Findings indicated that after eight MBSR sessions, the mean score of anxiety in the intervention group (7.9 ± 1.07) compared to the control group (13.79 ± 5.36), stress in the intervention group (9.26 ± 1.25) compared to the control group (18.13 ± 7.66), and depression in the intervention group (7.83 ± 1.05) compared to the control group (16.26 ± 11.06) were statistically significant, which indicates the effectiveness of counseling (*P* < 0.0001).

By comparing the pre- and post-test of each variable in each group, it was found that in the intervention group, the average score of anxiety decreased from 14.34 ± 5.35 before counseling to 9.7 ± 1.07 after counseling, i.e. counseling was able to reduce the level of anxiety (*P* < 0.0001). Also, the mean score of depression decreased from 15.11 ± 6.34 before counseling to 7.83 ± 1.05 after counseling (*P* < 0.0001). Regarding stress, we found that the average stress score decreased from 18.39 ± 5.47 before counseling to 9.26 ± 1.25 after counseling (*P* < 0.0001).

The total scores of the DASS questionnaire, which included stress, anxiety, and depression, were compared between the two groups. The comparison showed a significant difference between the two groups after intervention (*P* < 0.0001) (Table 3).

## Discussion

This study aimed to determine the effect of MBSR on anxiety, depression, and stress in women with bleeding leading to termination of pregnancy in the first trimester of pregnancy. The results showed a significant reduction in anxiety in the intervention group after counseling. Previous studies have shown that MBSR can reduce anxiety in pregnant mothers [15]. Also, an interventional study reported the effect of MBSR on reducing the symptoms of social anxiety in the dimensions of fear, avoidance, and physiological distress, which is in line with the findings of the present study. This may be due to the same consultation method used in both studies [16]. By using MBSR, people know themselves better by recognizing their strengths and weaknesses. They learn coping strategies, commitment, and acceptance, helping them move toward their desired goals, accept their mistakes and decisions without judgment, and deal with them promptly by identifying stressful stages and events, ultimately reducing stress and anxiety before they become depressive [14]. In a systematic review, Shi showed that mindfulness can reduce pregnancy-related stress and anxiety [17]. However, the reduction in anxiety in their control group was not consistent with the present study probably because their control group used books to increase information about pregnancy to help manage stress and some of them attended yoga classes.

In the present study, we see a significant reduction in stress after counseling in the intervention group. In one clinical trial, it was found that reducing stress based on mindfulness improves life orientation and reduces perceived stress in the experimental group. According to these results, mindfulness can create positive changes in happiness and well-being by combining vitality and the clear observation of experiences [18]. MBSR can also indirectly reduce stress by improving sleep conditions and sleep quality [19]. A study in Egypt showed the positive effects of mindfulness intervention and women's stress and mindfulness skills on managing the stressful stages of pregnancy [20]. First trimester bleeding and premature miscarriage are also stressful pregnancy events that we could be reduced by MBSR. A study conducted in 2018 by Krusche et al*.* Found that MBSR did not affect stress in pregnant women and that there was no significant difference between the intervention and control groups probably due to the online teaching method [21].

The present study's findings showed that MBSR was able to reduce depression after the termination of pregnancy in the first trimester. A 2019 study in China showed that MBSR was able to reduce pregnancy anxiety and stress but had no effect on depression [22]. However, a happiness-based research was able to reduce depression in women with recurrent miscarriages. A happiness training program improves patients' cognitive and emotional states. It allows patients to adopt a more positive attitude towards life events and respond to challenges optimistically by adapting to changing circumstances [23]. People with a conscious mind think about the past, analyze events to let go of bad habits, look at things in a new way, and see events as they are, increasing acceptance and satisfaction in their lives [24].

pregnancy can be a complex issue for many women and families. If it leads to pregnancy loss, it becomes an awful and irreparable experience that deeply affects their bodies and minds [25]. Sleep disorders and anxiety have been reported by 11.1% of women within the four weeks after the abortion. Psychologic healing after abortion takes five years, which is relatively long [26]. The feelings of loss remaining from previous pregnancies can last for years, and the healing process is complex and different for all women. The resulting anxiety may remain in couples who are planning for another pregnancy. It sometimes leads them to resort to destructive behaviors, which necessitates support and monitoring of couples after abortion. Abortion in women may be associated with increased risk of addiction to nicotine, alcohol, cannabis and other illegal drugs or suicidal thoughts and suicide attempts [27, 28]. Lack of counseling and follow-up leads to post-abortion anxiety disorder and increases and exacerbates mental disorders [26]. In mindfulness, a person learns how to deal with their ineffective and irrational thoughts and negative emotions. A mindful mind protects the person against stressors from psychological traumas by reducing irrational sensitivities. Self-control and continuous flow of awareness, being in the moment, and avoiding judgement increase long-lasting self-confidence and problem-solving skills. Mindfulness breaks the cycle of resistance to change. Self-reported body awareness, self-regulation, and well-being, positive changes in attitudes toward others, enlightened self-view, and development of critical consciousness are among the states and traits associated with mindfulness [29, 30].

considering that one of the practical and influential factors in improving the mental condition of women during pregnancy and afterward is social support and mindfulness counseling, this new and more practical method can be a basis for further research to be compared with other psychological methods. Social and familial support are different in different societies and cultures, and women are considered to be the guilty party and the cause of abortion in some. In some cultures, spontaneous abortion is considered a punishment for ungratefulness to God. Therefore, studies in communities with different cultures and religious beliefs can provide a better perspective for the provision of supportive counseling services by health care workers [31, 32]. In addition, the history of infertility, repeated abortions, chronic illness of the mother, the type of occupation of the father and mother, and the amount of family income can increase anxiety and stress in subsequent pregnancies [33, 34]. Therefore, studies can be conducted in people with different obstetric histories and demographic characteristics.

## Limitations

One of the limitations of the study was that it was not possible to check the rate and manner of family support of the women under study, and there is a possibility that the intervention group had better social and familial support. Another limitation of the study was that we could not categorize women based on the causes of spontaneous abortion, and women's attitudes may be different depending on the cause of abortion. The study location, which was a public hospital, also caused limitations. Because people of higher socio-economic status tend to go to private hospitals to receive care and treatment. Therefore, the study did not include all strata of society, and the results should be generalized with caution.

## Conclusion

According to the theoretical foundations and findings of the present study, it is clear that sessions of mindfulness training programs based on stress reduction can reduce anxiety, depression, and stress in women with bleeding leading to termination of pregnancy in the first trimester. Since the loss of pregnancy is an adverse event that leads to depression and anxiety and decreases quality of life, improving these women's mental health should be one of the priorities in the health system.

## Data Availability

Data are available from the authors upon reasonable request and with permission of Ethics Committee.

## Abbreviations

MBSR: Mindfulness-based stress reduction
EP: Ectopic pregnancy
PTSD: Post-Traumatic Stress Disorder
